# Neonatal care practices in sub-Saharan Africa: a systematic review of quantitative and qualitative data

**DOI:** 10.1186/s41043-018-0141-5

**Published:** 2018-04-16

**Authors:** Margaret Bee, Anushree Shiroor, Zelee Hill

**Affiliations:** 1 Concern Worldwide (UK), 13/14 Calico House, Clove Hitch Quay, London, SW11 3TN UK; 2 RESULTS UK, 31-33 Bondway, London, SW8 1SJ UK; 3 Institute for Global Health, University College London, 30 Guilford St., London, WC1N 1EH UK

**Keywords:** Newborn, Thermal care, Cord care, Breastfeeding, Sub-Saharan Africa

## Abstract

**Background:**

Recommended immediate newborn care practices include thermal care (immediate drying and wrapping, skin-to-skin contact after delivery, delayed bathing), hygienic cord care and early initiation of breastfeeding. This paper systematically reviews quantitative and qualitative data from sub-Saharan Africa on the prevalence of key immediate newborn care practices and the factors that influence them.

**Methods:**

Studies were identified by searching relevant databases and websites, contacting national and international academics and implementers and hand-searching reference lists of included articles. English-language published and unpublished literature reporting primary data from sub-Saharan Africa (published between January 2001 and May 2014) were included if it met the quality criteria. Quantitative prevalence data were extracted and summarized. Qualitative data were synthesized through thematic analysis, with deductive coding used to identify emergent themes within each care practice. A framework approach was used to identify prominent and divergent themes.

**Results:**

Forty-two studies were included as well as DHS data - only available for early breastfeeding practices from 33 countries. Results found variation in the prevalence of immediate newborn care practices between countries, with the exception of skin-to-skin contact after delivery which was universally low.

The importance of keeping newborn babies warm was well recognized, although thermal care practices were sub-optimal. Similar factors influenced practices across countries, including delayed drying and wrapping because the birth attendant focused on the mother; bathing newborns soon after delivery to remove the dirt and blood; negative beliefs about the vernix; applying substances to the cord to make it drop off quickly; and delayed breastfeeding because of a perception of a lack of milk or because the baby needs to sleep after delivery or does not showing signs of hunger.

**Conclusion:**

The majority of studies included in this review came from five countries (Ethiopia, Ghana, Malawi, Tanzania and Uganda). There is a need for more research from a wider geographical area, more research on newborn care practices at health facilities and standardization in measuring newborn care practices. The findings of this study could inform behaviour change interventions to improve the uptake of immediate newborn care practices.

## Background

Every year, approximately 2.9 million neonates die, which corresponds to 44% of deaths in children under 5 years of age [1, 2]. The first week of life is a particularly vulnerable period, with 73% of neonatal deaths occurring during this time [2]. Most of these deaths are preventable, but progress in reducing neonatal mortality has been slower than progress in reducing child or maternal mortality, meaning that neonatal deaths account for an increasing proportion of all child deaths [1]. Progress has been slowest in sub-Saharan Africa [1].

Interventions targeting labour, birth, the first week of life and small and sick neonates are likely to have the greatest impact on mortality [3]. Community-based interventions are important for reducing neonatal deaths, even where levels of facility deliveries are high [2]. After delivery, newborns should receive immediate newborn care, which includes thermal care (drying and wrapping, skin-to-skin care, delayed bathing), hygienic cord care and early initiation of breastfeeding. These practices can be promoted at the facility, community and household levels through a variety of channels including through post-natal home visits [3].

The consensus in the literature is that to be more effective, behaviour change interventions should be based on an understanding of the facilitators and barriers to behaviour change [4], and intervention strategies should reflect the local context [3]. Many programmes have neither the time nor resources to conduct substantial formative research to inform intervention design. In recent years, there have been a number of published and unpublished studies on immediate newborn care practices. In isolation, these studies provide important information for local policy makers, but they have less relevance for regional decision-making. Summarizing and synthesizing existing studies allow us to build a body of knowledge and explore the similarities and differences in practices and the factors that influence them between settings. This provides a foundation for future studies, highlights the level of context-specific adaptation behaviour change interventions may require and provides a baseline to help understand changes in behaviours over time. The aim of this paper is to systematically review published and unpublished literature from sub-Saharan Africa on key immediate newborn care practices. This review was done as part of a formative research study to inform a behaviour change trial on emollient therapy for newborns [5]. The review includes both quantitative survey data describing the prevalence of immediate newborn care practices and qualitative data exploring the factors that influence these practices.

## Methods

Recommended key immediate newborn care practices included in the review are thermal care (immediate drying and wrapping, skin-to-skin contact after delivery, delayed bathing), hygienic cord care and early initiation of breastfeeding. English-language studies reporting the prevalence of these immediate newborn care practices and/or exploring factors that influence them were identified by searching *MEDLINE*, *EMBASE*, *Web of Science*, *SCOPUS*, *Maternity and Infant Care*, POPLINE and ELDIS databases. A range of search terms and appropriate MeSH terms were used (see Table 1). In addition, the websites of Save the Children, Healthy Newborn Network, the Demographic and Health Surveys (DHS), UNICEF and WHO were searched. National and international academics, researchers and implementers were contacted for relevant unpublished reports. References from included articles and reports were hand-searched for additional studies. All retrieved references were entered into Endnote reference management software, and duplicates were removed.

**Table 1:** Search terms

*Concept 1 Newborns*: newborn OR newborns OR neonate OR neonates OR neonatal OR perinatal OR peri-natal OR postnatal OR post-natal OR baby OR babies OR preterm OR premature OR low birth weight OR low-birthweight OR LBW *Concept 2 Newborn care practices*: thermal regulation OR thermoregulation OR temperature OR thermal care OR bath* OR wash* OR wrap* OR warm* OR dry* OR wipe OR wiped OR wiping OR cover* OR clean* OR skin-to-skin OR skin to skin OR Kangaroo mother care OR cloth OR hypothermia OR vernix OR massage OR massag* OR application OR apply OR oil OR oils OR emollient OR emollients OR skin OR skincare OR skin-care OR topical OR Shea butter OR lotion OR powder OR paste OR umbilical OR cord OR umbilicus OR breastfeeding OR breast-feeding OR breastfe* OR breastmilk OR breast-milk OR breast milk OR prelacteal feeding OR pre-lacteal feeding OR prelactal feeding OR pre-lactal feeding OR colostrum OR colostrums OR feeding OR breath* OR asphyxia OR resuscit* OR danger-sign OR danger sign OR danger-signs OR danger signs OR care seeking OR care-seeking OR careseeking OR recognition *Concept 3 sub-Saharan Africa*: Africa OR sub-Saharan Africa OR Angola OR Benin OR Botswana OR Burkina Faso OR Burundi OR Cameroon OR Cape Verde OR Central African Republic OR Chad OR Comoros OR Congo OR DRC OR Democratic Republic of the Congo OR Democratic Republic of Congo ORCote d’Ivoire OR Ivory Coast OR Djibouti OR Equatorial Guinea OR Eritrea OR Ethiopia OR Gabon OR Gambia OR Ghana OR Guinea OR Guinea-Bissau OR Guinea Bissau OR Kenya OR Lesotho OR Liberia OR Madagascar OR Malawi OR Mali OR Mauritania OR Mauritius OR Mayotte OR Mozambique OR Namibia OR Niger OR Nigeria OR Reunion OR Rwanda OR Sao Tome and Principe OR Senegal OR Seychelles OR Sierra Leone OR Somalia OR South Africa OR Sudan OR Swaziland OR Tanzania OR Togo OR Uganda OR Zambia OR Zimbabwe	

Studies were included if they were published between January 2001 and May 2014, to ensure data still had relevance. Other inclusion criteria were that the paper reported primary data (i.e. were not a review or summary paper), was in English, included information on the selected newborn care practices, and reported data from sub-Saharan Africa.

Titles and abstracts were screened, and studies meeting the inclusion criteria were further screened by reading the full-text article. Once screened, the quality of the studies was judged. Quality was defined for the quantitative studies as the use of a probability sample, either of facilities or of community respondents. Surveys were excluded if recall periods were not reported or it was unclear how the data had been collected.

There is debate about assessing the quality of qualitative studies [6, 7], as important studies may be excluded for ‘surface mistakes’. We assessed qualitative studies using the tool from the Critical Appraisal Skills Program [8] but only excluded studies if there was evidence of a ‘fatal mistake’ that we felt would invalidate the findings [7]. Where a study was reported in more than one place, for example, in both a report and a published article, emphasis was given to the data in the journal article; however, the other source was also read thoroughly in case it provided more details of the methodology or results. Qualitative data were included if they used focus group discussions, in-depth interviews, key informant interviews or observations. Any concerns about exclusion or inclusion were resolved through a team discussion.

Quantitative prevalence data were extracted and summarized in tables for each care practice. Where DHS data that met the inclusion criteria were available, these were taken from the most recent DHS and used preferentially above other quantitative data. Qualitative data on the practices were synthesized through thematic analysis [7], with deductive coding used to identify emergent themes within each care practice. Coding was done by all authors and consisted of reading each article several times and manually applying codes in the margins of each article. These codes and related text and quotes were then extracted into Excel sheets using a framework approach [9], and the resulting Excel spreadsheet was used to identify prominent and divergent themes across the studies. These were entered into streamlined tables as the analysis progressed. A subsample of articles were coded by more than one person and consensus reached about the codes; this facilitated reflexivity and the identification of alternative codes. Quotes included in the results are from the articles and reports and are included to support and illustrate the key findings.

## Results

A total of 3994 references were identified from searches. Three thousand seven hundred twenty-two were excluded based on title or abstract. The remaining 272 articles were screened and 42 studies were included. Studies were excluded if they did not meet the inclusion criteria. Table 2 summarizes the included studies. The majority of studies were from Ethiopia, Ghana, Malawi, Tanzania and Uganda; with qualitative data from eight countries and quantitative data from nine countries. This is only a small proportion of the total countries in sub-Saharan Africa. Twenty-seven studies report qualitative data only, 10 report quantitative data only and 5 report both qualitative and quantitative data. DHS data was included from 33 countries on early breastfeeding practices. Data on other immediate newborn care practices was not available from the DHS.

**Table 2:** Included studies

Country	Qualitative data only	Quantitative data only	Qualitative and quantitative	Total
Ethiopia	6	2		8
Ghana	3		3	6
Malawi	4	2		6
Mali		1		1
Mozambique		1		1
Nigeria	1	1		2
Senegal	1			1
Sierra Leone		1		1
Tanzania	6	1		7
Uganda	4	1	2	7
Zambia	2			2
Total	27	10	5	42

Table 3 summarizes the quantitative survey methodologies and provides information on the level of facility deliveries; these ranged from 10 to 80% and were particularly low in Ethiopia and high in Malawi. The qualitative studies focused on community beliefs and practices; information on the reasons for facility practices was sparse. We report the findings divided by type of practice: thermal care, cord care and early breastfeeding practices.

**Table 3:** Location and sampling methods of surveys included in the review

Country	Date of survey	Percent facility delivery	Area covered	Sample
Ethiopia [19]	December 2008–January 2009	10%	Amhara, Oromiya, SNNP and Tigray Regions	Cluster sample of 600 women with babies 0–11 months with random walk at village level
Ethiopia [31]	January 2012	29%	Amhara, Oromiya, SNNP and Tigray Regions	Cluster sample of 218 women with babies 1–7 months, screening all women in each cluster
Ghana [11]	2006	Not reported	Brong Ahafo Region (6 districts)	All 635 women who had a live birth in a 2-week period identified through a demographic surveillance system, interviewed 1–28 days after delivery
Ghana [40]	April 2008–May 2009	Not reported	Brong Ahafo Region (6 districts)	All 9167 women who had a live birth between April 2008 and May 2009 in the control area of a community newborn trial identified through a demographic surveillance system, interviewed 1–28 days after delivery
Ghana [25]	July 2003–June 2004	27%	Brong Ahafo Region (6 districts)	All 2878 women who had a live birth in a 2-week period identified through a demographic surveillance system, interviewed 1–28 days after delivery
Malawi [22]	2007	80%	Mzimba district	Cluster sample of 300 mothers with children aged 0–23 months old, with random walk at village level
Malawi [32]	November–December 2007 (baseline data)	71%	Thyolo, Dowa, and Chitipa districts	Cluster sample of 900 women with children 0–12 months old, with random walk at village level
Mali [29]	2008	31%	Kayes, Koulikoro, Ségou and Mopti Regions (4 districts in each)	Cluster sample of 840 mothers who had a live birth in the last 12 months (no information on village level sampling)
Mozambique [20]	2008	47%	Nampula Province (3 districts)	Cluster sample of 517 women who had a live birth in the last 12 months (no information on village level sampling)
Nigeria [47]	2011	Not reported	Kware town in Sokoto state	Systematic sample of 179 mother-infant pairs who were breastfeeding or had done so in the last 2 years; houses were numbered by the study team
Tanzania [21]	2007	41%	Southern Tanzania (5 districts)	Census of 22,243 women who had a live birth in the last 12 months
Uganda [18]	2007	41%	Northern, Western, Central and Eastern regions and two divisions of Kampala (2 districts in each)	Cluster sample of 1136 households with children under 6 months with village guide listing eligible households at village level District hospital and one randomly selected health centre in each district included in a health facility assessment (39 facilities)
Uganda [23]	October–December 2011	43%	Masindi and Kiryandongo districts	Cluster sample of 928 lactating women with babies 0–5 months, with village guides leading team to eligible women
Uganda [24]	2007	46%	Iganga-Mayuge district	All 414 mothers with infants aged 1–4 months identified through a Demographic Surveillance System
Sierra Leone [33]	2008	Not applicable	Nationwide	All 38 public, private, mission and NGO hospitals providing maternal and child health services, a systematic random sample of 55 community health centers and a convenience sample of 52 health posts from all regions of the country

### Thermal care

The importance of keeping newborn babies warm was well recognized across the qualitative studies, with a shared belief that cold can make the baby sick: ‘If they don’t cloth her, and expose her to berd [cold], and do not prepare a warm place for her to sleep, they will put her at risk of illness’ [Ethiopian mother] [10]. Maintaining warmth by covering the baby in warm or extra clothing/wrappings, heating the room or keeping out drafts were common themes across a number of studies (Ethiopia, Ghana, Nigeria, Malawi, Tanzania, Zambia, Uganda) [10–18]. Keeping the baby inside was reported in Ghana, Ethiopia and Malawi [10, 11, 14], and exposing them to daily smoke was reported in Ethiopia [10].

#### Drying and wrapping after birth

Studies from six countries reported quantitative data on the timing of drying and wrapping the newborn (see Table 4). Two quantified timing while others referred to before the placenta was delivered or to soon/immediately after birth. Levels of early drying and wrapping were high in Mozambique and Ethiopia [19, 20], but sub-optimal in Ghana, Tanzania and Malawi [11, 21, 22]. Data from different areas of Uganda reported varying levels of drying and wrapping [18, 23, 24]. Data from Ghana illustrate that levels of early wrapping and drying can be influenced by place of birth, with levels higher for facility births [11].

**Table 4:** Quantitative and qualitative findings on drying and wrapping after birth by country

Country	Reference period	Percent wrapping and drying after delivery	Main reasons for delay
Ethiopia	Immediately	69% dried and wrapped [19]	Preoccupied with placenta, taboo to care for the baby until the placenta is buried [10]
Ghana	Within 5 min of birth	37% dried 29% wrapped Higher for facility births (42% dried and 36% wrapped) [11]	Preoccupied with placenta [11, 25], no one present is tasked with newborn care [11], waiting for cord cutting or bathing [11]
Malawi	Before placenta	57% wiped and wrapped [22]	No information
Mozambique	Before placenta	77% dried (home births) 84% wrapped (home births) District varied from 66 to 96% for drying and 71–98% for wrapping [20]	No information
Senegal	Not applicable	No information	Waiting for birth attendant to finish and for the baby to be bathed [26]
Tanzania	Within 5 min of birth	42% dried 27% wrapped [21]	Preoccupied with placenta, waiting for the baby to be bathed, impromptu delivery [27]
Uganda	Soon/immediately after delivery Before placenta	97% wrapped [18] 86% wrapped [24] 36% dried 49% wrapped [23]	No information

Qualitative data were available from Tanzania, Ghana, Senegal and Ethiopia (see Table 4). Common reasons for delayed drying and wrapping, reported from home deliveries in Tanzania, Ghana and Senegal, were birth attendants focusing on the mother until the placenta was delivered or because the birth attendant was busy caring for the mother [11, 25–27]: ‘Just after delivery the baby was put on a cloth between her mother’s legs to wait for the placenta to come out…….nobody concentrated on the baby until the placenta came out’ [Tanzanian mother] [27]. Other reasons for delays were waiting for the cord to be cut or for the baby to be bathed [11, 26, 27]: ‘…. we leave them there for 10 to 15 minutes….we wait until the birth attendant comes to look after them and we cover them after they have been washed’ [Sengalese mother] [26]. In Ethiopia, the focus was also on the mother until the placenta was delivered, but it was common for babies to be wrapped during this time [10, 28]: ‘If the placenta does not come out, they may wrap the newborn with cloth and put to the side….’ [Ethiopian father] [10].

Keeping the baby warm was the main reason for early drying and wrapping in Ghana, Ethiopia, Zambia and Uganda [10, 11, 17, 29, 30]: ‘Sometimes it is windy, and for a baby who has just been delivered, that is not good. So they quickly cut the cord, wrap it [the baby] in warm clothes and put it on the bed, so that it is kept warm, because the womb where it is coming from is warm’ [Zambian mother] [17].

#### Timing of first bath

Quantitative data from Ethiopia, Ghana, Mali, Tanzania and Uganda found that over 50% of newborns were bathed within 6 h of delivery (see Table 5) [11, 19, 21, 24, 29, 31]. Levels were particularly high, over 75%, in Ethiopia and the two West African countries [11, 19, 29, 31]. Practices were better in Malawi, with only 25% of newborns bathed within 6 h of delivery [32]. Data from Ghana and Ethiopia show that levels of early bathing can be high even in facilities [11], and an assessment of health facilities in Sierra Leone found that in over 50% of facilities, babies were bathed within 24 h of birth [33]. Qualitative data suggest that early bathing was uncommon in facilities in Malawi and Tanzania, partly because of a lack of water at facilities [13, 16, 32].

**Table 5:** Quantitative and qualitative findings on the timing of first bath by country

Country	Percent bathed within 6 h of birth	Main reasons for early bathing
Ethiopia	66% within 6 h [19, 31]: 81% for home and 58% for facility births [31]	Clean the baby, remove odor and make the baby stronger [10]
Ghana	82%: 93% for home and 77% for facility births [11]	Stop body odor later in life [11, 25], shape head, make the baby feel clean, help the baby sleep [11]
Malawi	25% within 6 h [32] 52% within 24 h [22]	Remove dirt and smells [14, 16], make the baby beautiful [14], refresh the baby and help sleep [16], remove vernix (linked with sperm) [16, 37]
Mali	78% [29]	No information
Senegal	No information	Remove blood, sperm and impurities, make baby comfy, stop body odor later in life, stop baby getting sick [26]
Sierra Leone	53% of facilities routinely bathe the baby within 24 h (health facility survey) [33]	No information
Tanzania	59% [21]	Remove the dirt and vernix (linked with sperm) [27], make baby strong and help baby to cry [36]
Uganda	56% [24]	Remove dirt and odor [30, 34, 35], make healthy and hygienic [18], make baby comfy [34], remove vernix (linked to sex) [30], make baby clean for visitors [34]

Reasons for early bathing were available from qualitative studies from Malawi, Senegal, Ghana, Uganda, Ethiopia and Tanzania (see Table 5), with removing dirt, blood and other fluids being the main reason for the practice [10, 11, 14, 16, 18, 26, 27, 30, 34, 35]. This was linked to making the baby refreshed and comfortable (Ghana, Malawi, Senegal and Uganda) [16, 21, 26, 34], encouraging sleep (Malawi and Ghana) [11, 16] and improving the health/strength of the baby (Senegal, Ethiopia, Tanzania, Uganda) [10, 18, 26, 36]: ‘…the blood that coagulates on its body contracts its muscles.…. Until it has been washed, the baby will spend all its time crying, and that alone can make it sick.’ [Senegalese mother] [26].

Another key reason for early bathing was linked to smell. Mothers in East and Central African countries were concerned that the birth fluids smell bad [10, 14, 16, 30, 34], whilst those in West Africa were concerned about preventing body odour in later life [11, 25, 26]: ‘It’s unthinkable to leave it even one hour after the birth without washing it…. the liquid on the body penetrates into the skin, it stays there forever and causes a foul smell’ [Senegalese mother] [26]. An obvious vernix caseosa was also a reason for early bathing, with beliefs about the vernix being sperm in the Malawi, Tanzania, Uganda and Senegal [16, 26, 27, 30, 37] resulting in social pressure to remove a visible vernix quickly: ‘..if the baby is born with white things on the skin means the baby is dirty with sperm, it means that women will not let the baby remain with those white things on skin because they will be feeling shy when people will come to see the baby’ [Tanzanian mother] [27].

Delayed bathing, when it did occur, was linked to the need to keep the baby warm (Ethiopia, Ghana and Malawi) [10, 11, 14] and occurs, for example, if the weather was cold at the time of delivery or if the baby was delivered at night [11, 36, 37]: ‘If you deliver around 10 pm they wipe off the birth fluid and wrap the baby for the night….The baby is not bathed in the night because during that time the weather will be cold’ [Ghanaian mother] [11]. In these sites, health worker advice was also reported a reason for delayed bathing [11, 13, 16, 27].

All of the qualitative studies with information on the frequency of bathing (Ethiopia, Ghana, Malawi, Nigeria, Tanzania and Uganda) suggest that frequent bathing of newborns is the usual practice [10, 12–14, 25, 27, 34, 38]. Reasons were variable including to prevent odours later in life (Ghana and Nigeria) [12, 25, 38], keep the baby cool and/or comfy (Malawi and Tanzania) [14, 27], help the baby sleep (Ghana and Malawi) [14, 25, 38] or keep the baby clean and heal sores/prevent sickness (Ghana and Ethiopia) [10, 38].

#### Skin-to-skin contact after delivery

Skin-to-skin (STS) contact was extremely rare in all countries for which quantitative data was available [11, 21, 24, 29, 31], including for facility deliveries [11, 32]. Barriers to skin to skin contact are listed in Table 6; however, only one study reported on actual experiences of women who had tried skin-to-skin contact [39], with other studies reporting hypothetical barriers. Concerns around transmission of disease and the mother or baby being dirty were reported in Uganda, Malawi and Senegal [16, 26, 30, 35, 39]: ‘If the baby is born, it is very soft, so to me it is not right as the mother may be sick and can transmit disease to the baby. It can get the disease through the sweat and it is better the baby is placed by side and covered with a cloth’ [Ugandan father] [30]. In one of the Ugandan studies, disease transmission was linked to ‘bad heat’ associated with delivery that needs to be washed off before the mother carries the baby [35]. Concerns around skin-to-skin contact disrupting the mother’s ability to rest, causing exhaustion or being unfeasible because the mother was in pain, were reported in Malawi, Uganda and Tanzania [16, 27, 39] and hurting the baby in Tanzania and Uganda [27, 39]. A lack of opportunity for skin-to-skin contact, due to activities taking place after birth, was reported in Ghana and Tanzania [27, 38]: ‘The baby is given to the mother only when it has been fully attended to and wrapped’ [Ghanaian mother] [38].

**Table 6:** Quantitative and qualitative findings on skin-to-skin contact after delivery by country

Country	Percent placed skin-to-skin (STS) after delivery	Barriers to skin-to-skin after delivery
Ethiopia	13% had STS on the day of delivery (8% for home and 26% for facility) [31]	No information
Ghana	8% had any STS in the first 24 h (10% for home and 6% for facility births) [11]	Few opportunities due to other activities [11, 38]
Malawi	No information	Exhausts mother Mother not clean enough for breastfeeding [16]
Mali	2% placed on the mother’s chest* [29]	No information
Senegal	No information	Mother’s sweat could pass illness [26]
Tanzania	1% placed on the chest after the cord is cut (home births) [21]	May hurt the babies’ cord, chest or bones Few opportunities due to other activities Mother is in pain/has problems after birth STS is not practiced at the facility STS is not necessary [27]
Uganda	2% had STS* [24]	Baby/mother is dirty and could transmit disease [30, 35], particularly HIV through the umbilicus [39] May hurt the cord [39] Baby would get cold if not wrapped [30] Difficult for mother to rest and invasion of privacy [39]

*Time not given

The mothers who tried skin-to-skin contact in Uganda reported several positive aspects including having immediate access and feeling close to the baby and starting breastfeeding quickly [39]: ‘I think it teaches us to start loving our baby from the very beginning….The pain had made me hate the baby. I even told the nurses on the labour ward I do not think I will have love for this child. However, to my surprise after telling them, they still place the baby on my chest and somehow the affection came naturally.’ [Ugandan mother] [39].

#### Hygienic cord care

Cord care practices were highly variable (see Table 7), with over 90% of babies having something applied to the cord in two West African studies [29, 40], but lower levels were found in other countries [19–21, 31]. Data from two Ugandan studies conducted in different areas and several years apart found contrasting results, suggesting that there may be regional differences in cord care, that there has been a change in practice over time or that the results are not comparable due to methodological differences [23, 24]. Quantitative data reported that the types of substances commonly applied to the cord varied between countries: shea butter in Ghana, hospital medicine/spirit in Ghana and Mozambique, butter in Ethiopia, cooking oil or herbs in Tanzania and Mozambique and powder, salt water or herbs in Uganda [21, 23, 31, 40, 41].

**Table 7:** Quantitative and qualitative findings on cord care by country

Country	Percent applying substances to the cord	Main reasons for application
Ethiopia	32% used something to dress the cord [19] 35% applied something immediately after the cord is cut (27% for home and 53% for facility) 17% applied butter [31]	Help the cord dry, prevent wind from entering the baby, prevent pain and bad smell [10]. Soften and keep the cord moist, protect from wounds/infections and help heal [42]
Ghana	92% applied something (home deliveries) 31% applied hospital medicine 47% applied shea butter [40]	Make the cord drop fast to reduce discomfort for mother and baby [25, 40] and make baby human [25] Keep soft and wet to heal internal sores [40], stop bad smells [25], reduce illness and death [40]
Malawi	No information	Make the cord drop fast to shorten the confinement period, keep the cord soft/moist to reduce bleeding and infection [16]
Mali	90% applied something [29]	No information
Mozambique	15% applied something (home delivery) [20], substances varied by district and included herbs, spirit and oil	No information
Nigeria	No information	Prevent infection [12]
Senegal	No information	Dry the cord, prevent wind and water from entering the baby causing sickness [26]
Tanzania	28% applied something 8% applied traditional medicine 7% applied oil [21]	Make cord dry [43], drop fast to reduce period of vulnerability [44] and keep the baby healthy [43]
Uganda	49% applied something 22% applied powder 11% applied salt water [24] 79% applied something 62% applied powder 7% salt water 6% herbs [23]	Make the cord drop fast so the mother can return to chores [30, 34], to reduce period of vulnerability and make baby human [35] and stop afterbirth pains [34] Dry the cord [18, 34], stop the cord going bad/heal the cord/prevent infections [18, 34, 35], help the baby sleep [30]
Zambia	No information	Dry the cord to make it drop fast and shorten the period of vulnerability, reduce afterbirth pains Make the cord soft/moist to prevent cracking and bleeding, prevent and treat infection [45]

Applying substances to the cord was most commonly done to prevent infections or help the wound heal (Ethiopia, Tanzania, Uganda, Malawi, Senegal, Zambia, Nigeria, Ghana) [12, 18, 25, 26, 30, 34, 35, 40, 42–45]. There was a desire for the cord to drop off quickly in several countries, and this was one of the main reasons for applying substances in Malawi, Uganda, Ghana, Zambia and Tanzania (see Table 7) [16, 25, 30, 34, 35, 40, 43–45]. ‘If it doesn’t drop fast, then there will be dirty air going through the umbilical and this will cause problem inside the baby. It’s better that it drops off quickly so that it closes’ [Zambian mother] [45]. Substances were applied to dry the cord, usually linked to helping it drop off (Ethiopia, Uganda, Senegal, Tanzania) [10, 18, 26, 34, 43] or to keep the cord moist or soft so it does not bleed, get infected or hurt the baby (Malawi, Uganda, Ethiopia, Zambia, Ghana) [16, 30, 40, 42, 45].

The timing of the cord dropping had great significance in several studies, because it was considered to be the end of the postpartum seclusion period, after which mothers are free to move outside their homes (Uganda, Malawi and Zambia) [16, 18, 30, 34, 35, 37, 45]. ‘There is no problem to simply put nothing on the cord. The cord will still fall but it will take a long time the mother will get tired of waiting in chikuta (seclusion)’ [Malawian mother] [16]. In several studies, it was the time when the baby is believed to become human or less vulnerable to illness or to people with bad intentions (Tanzania, Uganda, Malawi, Zambia and Ghana) [16, 25, 35, 37, 44, 45]. The dropping of the cord was also linked to the mother and baby become free of afterbirth pain (Zambia, Uganda and Ghana) [25, 34, 40, 45].

In Ethiopia, Ghana, Uganda, Senegal and Zambia, a theme emerged around the cord being a link between the external world and the inside of the baby [10, 25, 26, 35, 40, 45]. This mostly led to substances being applied to ensure the orifice was blocked so that wind, water or other environmental dangers did not enter the baby and cause illness [10, 26, 35], but in Ghana, this belief led to substances being applied to keep the channel open to help internal sores heal [25, 40]: ‘The shea butter helps the surface of the sore to be wet so that it won’t close up….If it closes up it will look like it is healed but there will be some sore inside the cord’ [Ghanaian mother] [40].

#### Early breastfeeding practices

Breastfeeding was the only immediate newborn care practice with quantitative data available from the Demographic and Health Surveys (DHS) [46]. Data that met the inclusion criteria were available for 33 sub-Saharan African countries and are presented in graphical form for ease of comparison. As the DHS data are comparable across sites, we have not reported quantitative prevalence data from other sources for this section.

#### Initiation of breastfeeding

**Fig. 1:**
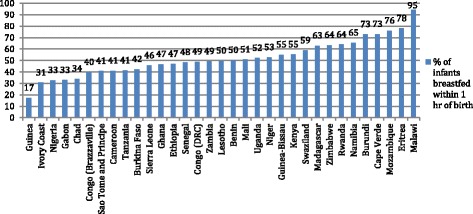
Initiation of breastfeeding within 1 h of birth in sub-Saharan Africa. DHS data on initiation of breastfeeding within 1 h of birth

Levels of early initiation ranged from 17 to 95% (see Fig. 1), with almost half of the countries reporting that less than 50% of newborns initiated breastfeeding within an hour. Only seven countries had data disaggregated by region, and all except Malawi showed large variations within the country. For example, early initiation of breastfeeding ranged from 18 to 75% across regions in Senegal, 27–70% in Uganda and 38–67% in Ethiopia.

Only a Ghanaian and a small Nigerian study reported quantitative data on reasons for delayed initiation of breastfeeding, with beliefs about colostrum (Nigeria) and perceived lack of milk (Ghana and Nigeria) being the main reasons for the delay [25, 47]. These were also common reasons in qualitative data, with a belief that milk does not arrive until a few days after birth (Ethiopia, Tanzania, Ghana, Nigeria) [10, 12, 15, 25, 36, 47–49] or that the colostrum was dirty or harmful and should not be fed to the child (Uganda, Tanzania, Ghana, Ethiopia and Nigeria) [12, 15, 34, 47, 48, 50]: ‘She didn’t give breast milk because there was none in her breast she squeezed and realized that nothing at all was coming out. She gave tinned milk for 2 days. The breast milk came in the evening of the second day.’ [Ghanaian mother] [48].

Several studies reported that the baby needing to sleep/rest after delivery or not showing signs of hunger were reasons for delayed initiation of breastfeeding (Ethiopia, Malawi, Senegal, Ghana, Zambia, Nigeria, Tanzania, Uganda) [10, 12, 14, 17, 26, 35, 36, 48]: ‘…there is a newborn who comes hungry so that one is breastfed there and then, but there are others who come when they are full so those ones don’t breastfeed there and then because the mother had eaten before giving birth’ [Ugandan traditional birth attendant (TBA)] [35].

Delayed initiation of breastfeeding was also linked to post-delivery activities, such as the mother and/or baby needing to be bathed or the mother needing to rest after delivery (Malawi, Tanzania, Zambia, Ghana, Ethiopia) [14, 16, 17, 36, 48, 50]: ‘The blood during birth is very dirt so we allow the mother to bath before breastfeeding. It is unhygienic to breastfeed before bathing’ [Malawian respondent] [14] (Table 8).

**Table 8:** Quantitative and qualitative findings on delayed initiation of breastfeeding by country

Country	Reasons for delayed initiation (quantitative)	Reasons for delayed initiation of breastfeeding (qualitative)
Ethiopia		Lack of milk [10, 49] Baby not ready to feed [10] Colostrum dirty/unhealthy for baby [50] Mother and baby need to bath [50]
Ghana	84% lack of milk 11% baby refused 4% mother or child ill [25]	Lack of milk [25, 48] Mother and baby need to bath [48] Beliefs about colostrum [48] Baby sleeping and/or not showing signs of hunger [48] Milk bitter the first few days Mother needs to undergo cleansing ritual the first few days [51]
Malawi		Mother and/or baby need to bath [14, 16] No signs of hunger [14]
Nigeria	68% colostrum dirty or harmful 14% lack of milk 13% mother or child ill [47]	Lack of milk Baby needs rest [12] Colostrum dirty and harmful [12, 47]
Senegal		Baby sleeping [26]
Tanzania		Lack of milk [15, 36], colostrum is not suitable to feed [15] Baby did not cry [36] Mother and baby need to bath [36] Mother is in pain after delivery [36]
Uganda		Lack of milk or colostrum is harmful [34] No signs of hunger [35]
Zambia		Mother is in pain post-delivery and needs rest Baby is sleeping and/or not showing signs of hunger [17]

When it did occur, reasons for early initiation of breastfeeding varied across studies and included keeping the baby warm and strong, helping the milk flow (Ghana) [48, 51] and facilitating the expulsion of the placenta (Ethiopia) [10], because the baby cried or would be hungry after delivery (Tanzania, Malawi) [14, 36], and because delayed initiation was associated with being HIV positive (Tanzania) [44], ‘She said she would like to breast feed her baby immediately after birth, when the baby is born must be hungry so you have to breastfeed the baby, or the baby will be crying all the time.’ [Tanzanian mother] [36].

In some studies, delayed initiation of breastfeeding was associated with babies being fed substitutes such as water, sweetened water, water mixed with bread, honey and butter (Ethiopia, Uganda, Ghana, Tanzania and Nigeria) [10, 12, 13, 15, 25, 34–36, 47, 48, 50] or milk products such as animal, formula or evaporated milk (Ethiopia, Tanzania, Ghana and Nigeria) [25, 36, 47, 48]**:** ‘After delivery I could not feed my child because there was nothing in my breasts. I just mixed some sugar with warm water to feed it’. [Tanzanian mother] [15]. In Ghana, substitutes were only given if initiation of breastfeeding was delayed for over a day [48].

Prelacteals, most often herbal mixtures, were given to open/clear the bowels or airways (Uganda and Nigeria) [12, 18, 35], soothe the babies’ throat (Ethiopia) [49] or protect against illness (Malawi, Senegal) [26, 52]. Among Muslims in Senegal, Nigeria and Ethiopia, water or milk imbibed with the Koran was also given [26, 47, 50]: ‘Here, no baby feeds from its mother’s breast as soon as it is born, without first drinking ‘toxantal’ water. Otherwise, the child is liable to be more like the pagans and grow up stupid.’[Senegalese respondent] [26].

**Fig. 2:**
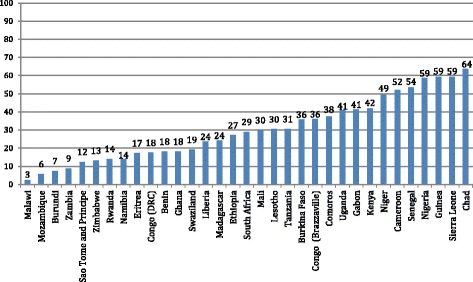
Prelacteal feeding (%) in the first 3 days of life in sub-Saharan Africa. DHS data on prelacteal feeding in the first 3 days of life

Figure 2 shows the levels of prelacteal feeding, measured by the DHS, for 33 sub-Saharan African countries. Levels of prelacteal feeding were over 40% in about a third of countries and under 10% in only four countries. The practice appears to be particularly prevalent in a number of West African countries.

## Conclusion

The quantitative surveys reported considerable variation in the prevalence of immediate newborn care practices between countries, and in some cases, there was considerable variation within countries. The exception was skin-to-skin contact, which was universally low. Some of the variation could be due to data collection differences, as different surveys collected data in different ways, asked women different questions, reported different reference periods and were collected at different time points. For example, the reference period for wrapping and drying the baby varied from a specific time (within 5 min), a specific event (before or after the placenta was delivered) to a more subjective measure such as soon/immediately after delivery. Information about what was put on the cord rarely had a reference period.

Recently, there have been calls for standardization in measuring newborn care practices [53, 54]. This review highlights the importance of this to improve the comparability of data. The only immediate newborn care practice with comparable data available from the DHS was early breastfeeding. Studies have found that measures of newborn care practices have varied validity [55], and the need to further test and validate measures has been recognized [54]. Qualitative research could help improve question formulation. A study using cognitive interviewing techniques in Ethiopia found that when women were asked about initiating breastfeeding, they perceived that they were being asked about when the baby received breastmilk, rather than when they were put to the breast [56]. A study, by the same authors, identified the influence that data collector probes may have on data validity [57]. Using cognitive interviewing to modify how questions are asked, and providing standard probes for data collectors, could improve question validity.

In contrast to the variation in the reported prevalence of immediate newborn care practices, the reasons for these practices were remarkably similar across studies—despite contextual differences. The need to keep newborns warm was well recognized, and this belief may make interventions promoting thermal care practices more acceptable. Other beliefs that were similar across studies were delayed drying and wrapping because the birth attendants focused on the mother; bathing newborns soon after delivery to remove the dirt and blood; negative beliefs about the vernix; applying substances to the cord to make it drop off quickly; and delayed breastfeeding because of a perception of a lack of milk or because the baby needs to sleep after delivery or does not show signs of hunger. These findings can be used to guide future studies. The findings could also be useful for intervention implementers, for example it is now recommended that chlorhexidine is applied to the cord for infants born at home in settings with high neonatal mortality [58]; it would be useful for implementers to monitor perceptions on whether application affects the time it takes the cord to drop off and whether this influences utilization.

Most of the studies included in this review came from five countries (Ethiopia, Ghana, Malawi, Tanzania and Uganda), representing only a small proportion of African countries, and there is a need for more research from a wider geographical area. The number of countries included was small; but they were from varied settings. The inclusion of studies from disparate settings enhances qualitative synthesis, and we were able to compare and contrast findings (reciprocal and refutational translation) and fiund that key themes were reciprocal across studies [59]. There was a focus on home births among the qualitative data, and few of the surveys disaggregated data by place of birth. With the increase in facility births that is occurring in the region, more research is needed to understand why immediate newborn care practices do or do not happen for facility births and whether the consequences of the practices are same for births that occur at home vs. births that occur in health facilities.

## Abbreviations

DHS: Demographic and Health Surveys
STS: Skin-to-skin contact
TBA: Traditional birth attendants
